# Linkage analysis of high myopia susceptibility locus in 26 families

**Published:** 2008-12-30

**Authors:** Sandrine Paget, Sophie Julia, Zulma G. Vitezica, Vincent Soler, François Malecaze, Patrick Calvas

**Affiliations:** 1 Inserm, U563, Centre de Physiopathologie de Toulouse Purpan, Toulouse, France; 2 Université Toulouse III Paul-Sabatier, UMRs563, Toulouse, France; 3 CHU de Toulouse, Hôpital Purpan, Service de Génétique Médicale, Toulouse, France; 4 CHU de Toulouse, Hôpital Purpan, Service d’Ophtalmologie, Toulouse, France

## Abstract

**Purpose:**

We conducted a linkage analysis in high myopia families to replicate suggestive results from chromosome 7q36 using a model of autosomal dominant inheritance and genetic heterogeneity. We also performed a genome-wide scan to identify novel loci.

**Methods:**

Twenty-six families, with at least two high-myopic subjects (ie. refractive value in the less affected eye of −5 diopters) in each family, were included. Phenotypic examination included standard autorefractometry, ultrasonographic eye length measurement, and clinical confirmation of the non-syndromic character of the refractive disorder. Nine families were collected de novo including 136 available members of whom 34 were highly myopic subjects. Twenty new subjects were added in 5 of the 17 remaining families. A total of 233 subjects were submitted to a genome scan using ABI linkage mapping set LMSv2-MD-10, additional markers in all regions where preliminary LOD scores were greater than 1.5 were used. Multipoint parametric and non-parametric analyses were conducted with the software packages Genehunter 2.0 and Merlin 1.0.1. Two autosomal recessive, two autosomal dominant, and four autosomal additive models were used in the parametric linkage analyses.

**Results:**

No linkage was found using the subset of nine newly collected families. Study of the entire population of 26 families with a parametric model did not yield a significant LOD score (>3), even for the previously suggestive locus on 7q36. A non-parametric model demonstrated significant linkage to chromosome 7p15 in the entire population (Z-NPL=4.07, p=0.00002). The interval is 7.81 centiMorgans (cM) between markers D7S2458 and D7S2515.

**Conclusions:**

The significant interval reported here needs confirmation in other cohorts. Among possible susceptibility genes in the interval, certain candidates are likely to be involved in eye growth and development.

## Introduction

Myopia is currently divided into low to moderate myopia (refractive values between −0.5 and −5 diopters [D]) and high myopia (beyond −5 D). The prevalence of myopia varies moderately in Western countries, ranging from 16% in Australia and 18% in the Netherlands, to an average value of 25% in the USA in adults between 40 and 80 years old [1]. The Asian population seems to be more affected than Western populations. The prevalence of myopia ranges from 16% in Australia and 18% in the Netherlands to 25% in the United States in adults aged 40–80 years [1] to much higher values in Eastern Asian countries. Over 38% of urban Singaporean Chinese adults [2] and up to 80% of teenagers (16–18 years old) in urban Taiwan [3] are affected. For high myopia, the prevalence is 4.5% in populations of Western European origin [1] as compared to the 8%–9% [2,4] observed in Eastern Asian adults over the age of 40. Laser refractive surgery as a myopia-related cost was estimated to be 4.6 billion dollars for the United States alone in 1990 [5]. Stambolian et al. [6] estimated that by 2005 this cost had doubled.

Increasing prevalence and associated health costs [5,6] make myopia an important public health problem [7,8]. High myopia predisposes patients to premature cataracts and an increased risk for retinal detachment, glaucoma, macular degeneration, and blindness [9]. It is one of the major causes of legal blindness worldwide [10,11]. No one certain cause of myopia has yet been identified. Several family and twin studies have shown the role of genetics in the etiology of myopia [12-16]. Different inheritance models have been proposed [14,17]. It is mostly thought that myopia results from interactions between genetic and environmental factors. For example, close vision and near-work activities increase myopia prevalence (for review, see Gilmartin [18] and Morgan and Rose [19]).Different chromosomal localizations for high myopia have been reported in linkage analyses over the last decade. To date, syndromic and isolated forms of X-linked high myopia have been respectively associated with Xq28 [20] and Xq23–25 [21,22]. Larger autosomal regions have been reported for non-syndromic high myopia. Some were identified in single large families [23-26] or in subsets of families [27-30]. Low and moderate myopia has also been linked to different regions [31-34]. Candidate genes have also been suggested [35-39]. For instance, the 5′ region of *HGF* (hepatocyte growth factor) in mouse [40] and man may contain a polymorphism associated with early-onset extreme myopia (beyond −10 D) in the Han Chinese population [41].

Our previous studies analyzed the inheritance of high myopia, suggested an autosomal dominant model with weak penetrance [42], and found a suggestive linkage to high myopia on chromosome 7q36 [28]. To confirm the previous linkage peak and to look for other loci involved in high myopia, a novel study was conducted in an extended population. We were able to collect nine additional families. In this study, our goal is to perform a genome-wide scan (GWS) on these nine new families as well as the combined linkage analysis on the full sample of families.

## Methods

### Subjects

Volunteers received information about the study in agreement with the Helsinki Declaration principle. Participants were included according to the French laws governing participation in biomedical research and with approval of the local ethics committee. Each participant provided written, informed consent. Families of high myopia were enrolled through a proband affected with non-syndromic high myopia (refractive value, RV≤ −5 D, axial length>26 mm). The family was retained for the study if it included at least two high myopic members and if at least three members on two generations agreed to participate. Exclusion criteria were myopia in prematurity retinopathy, Marfan’s syndrome, Stickler’s syndrome, Wagner’s disease, retinitis pigmentosa, corneal dystrophy, keratoconus, myopia secondary to cataract, or any developmental genetic disorder. In addition, families with unilateral high myopia and families with X-linked compatible high myopia were excluded.

Twenty-six families were included in this study (nine new families and 17 previously studied families [28]). The new families (16, 18, 20–26) were constituted by 136 persons (34 high myopic subjects). Among the 17 previously studied families, five (1, 4, 10, 12, and 14) had a total of 20 new participants with one to eleven additional individuals per family. All the members in these families under 20 years of age at the time of the first evaluation [28] were phenotypically re-evaluated, and it was found that a total of five persons in four families (individual 15 [ID15] in family 5 [F5], ID4 in F11, ID6 in F17, ID4 and ID7 in F19) who had moderate myopia became high myopic. Age at recruitment was between 5 and 95 years old.

### Clinical examination

Refractive values (RVs) were measured using standard autorefractometry after dilation. In patients under 16 years old, three instillations of 1% cyclopentolate at 0 min, 5 min, and 10 min were performed and RVs were measured 45–60 min after the last instillation. In patients over 16 years old, dilation was induced with 1% tropicamide at 0 min, 5 min, and 10 min, and the RV measured 30–45 min later. Tropicamide (1%) was instilled every 5 min three times, and then measurement is performed. Axial length was evaluated by A-scan ultrasonography. A total of three readings were taken for each eye, and the average value was recorded. Astigmatism was assessed by autorefractometer.  The included patients had minimal astigmatism, indicating that bias was avoided. Each proband had a complete ocular examination including visual acuity, intraocular pressure, and fundus, to confirm that the high myopia was a primary finding and in completion of a general physical examination to avoid recruitment of syndromic myopia patients. For the probands’ relatives, ophthalmologists of their choice were asked to examine subjects according to the standard protocol above. In the case of cataract surgery, only the ocular measurement before surgery was considered.

### Genotyping

DNA extraction from venous blood was performed according to standard phenol-chloroform extraction procedure [43]. For genotyping, four DNA size references were added to standardize size-calling between runs according to the manufacturer’s procedure (Applied Biosystems, Foster City, CA). The average spacing distance between markers was 9.1 centiMorgan (cM), and the average heterozygosity was 0.78. The reduction of the detected chromosomal locus was made by genotyping of highly heterozygous microsatellites as per the UniSTS (NCBI) and GDB databases.

Amplifications of microsatellites were performed in 10 µl total volume, with 1X GoTaq PCR buffer (Promega, Madison, WI), 2.0–2.5 mM MgCl_2_, 200 µM each dNTP, 250 nM specific primer to each marker, 0.28 U/µl GoTaq DNA polymerase (Promega), and 25 ng of genomic DNA. Fluorescent labeled amplification products were electrophoresed on an ABI PRISM® 3100 Genetic Analyzer with GeneScan 500HD ROX standard size label (Applied Biosystems) and analyzed with Genescan 3.5 software (Applied Biosystems). Alleles were analyzed with Genotyper 3.6 program (Applied Biosystems).

### Statistical analyses

Familial relationship inconsistencies and genotyping error checking and cleaning were performed with the Merlin 1.0.1 program [44,45]. Marker allele frequencies were estimated from the founders of the pedigrees. Sex-average genetic distances were taken from the Marshfield Center for Medical Genetics. The order consistency was compared to the NCBI physical map (Build 36.2). The effects of ethnic origin, sex, and age were compared between affected and unaffected individuals using a Fisher’s exact and student’s *t*-tests with a level of significance below the p value of 0.01. In this study, RV is a spherical equivalent (the correlation between RV and spherical equivalent was 0.76 and 0.85 for the right and the left eye, respectively). The RV correlation between right and left eye was equal to 0.95. The RV mean was used for status qualification and as the trait in quantitative trait loci (QTL) analyses. Subjects with a RV mean of less than or equal to −5 D were considered to be affected. The others persons were classified as unaffected.

Multipoint parametric linkage (PL) and non-parametric linkage (NPL) analyses were performed under multiple models with Genehunter 2.0 [46] and Merlin 1.0.1 programs [44]. A total of eight parametric models with different inheritance model, genotype penetrance, and phenocopy rates were tested with a frequency of the susceptibility allele of 0.013 (Table 1). For each model, the hypothesis of genetic heterogeneity was also tested, and Heterogeneity Log of the Odd (HLOD) was calculated.

**Table 1:** Parametric models used in the parametric multipoint genome-wide linkage analysis.

**Phenocopy rates and penetrances**				
	**DD**	**Dd**	**dd**	**Model**
Autosomal recessive models	0	0	0.58	Model 1
	0	0	0.9	Model 2
Autosomal dominant models	0	0.58	0.58	Model 3
	0	0.9	0.9	Model 4
Autosomal additive models	0.1	0.58	0.58	Model 5
	0.1	0.9	0.9	Model 6
	0.2	0.58	0.58	Model 7
	0.2	0.9	0.9	Model 8

Based on the postulation of a single, two-allele gene in which “d” would be the disease-causing allele, eight parametric models have been tested. Models one and two assume an autosomal recessive mode of inheritance. Models three and four assume an autosomal dominant mode of inheritance. Models six to eight assume autosomal additive transmission as per Chen et al. [30]. In all models, penetrances of the genotype of 0.58 and 0.90 were used according to those reported respectively by Naiglin et al. [28] or Young et al. [23,27]. In addition, models five to eight consider ten or twenty percent phenocopy rates.

Myopia was also treated as a quantitative trait. The RV mean was transformed to reach normality [47]. A QTL analysis was conducted with Merlin 1.0.1 using the regression option. The sex, age, and ethnic origin were included as covariates.

## Results

### Population characteristics

**Figure 1:**
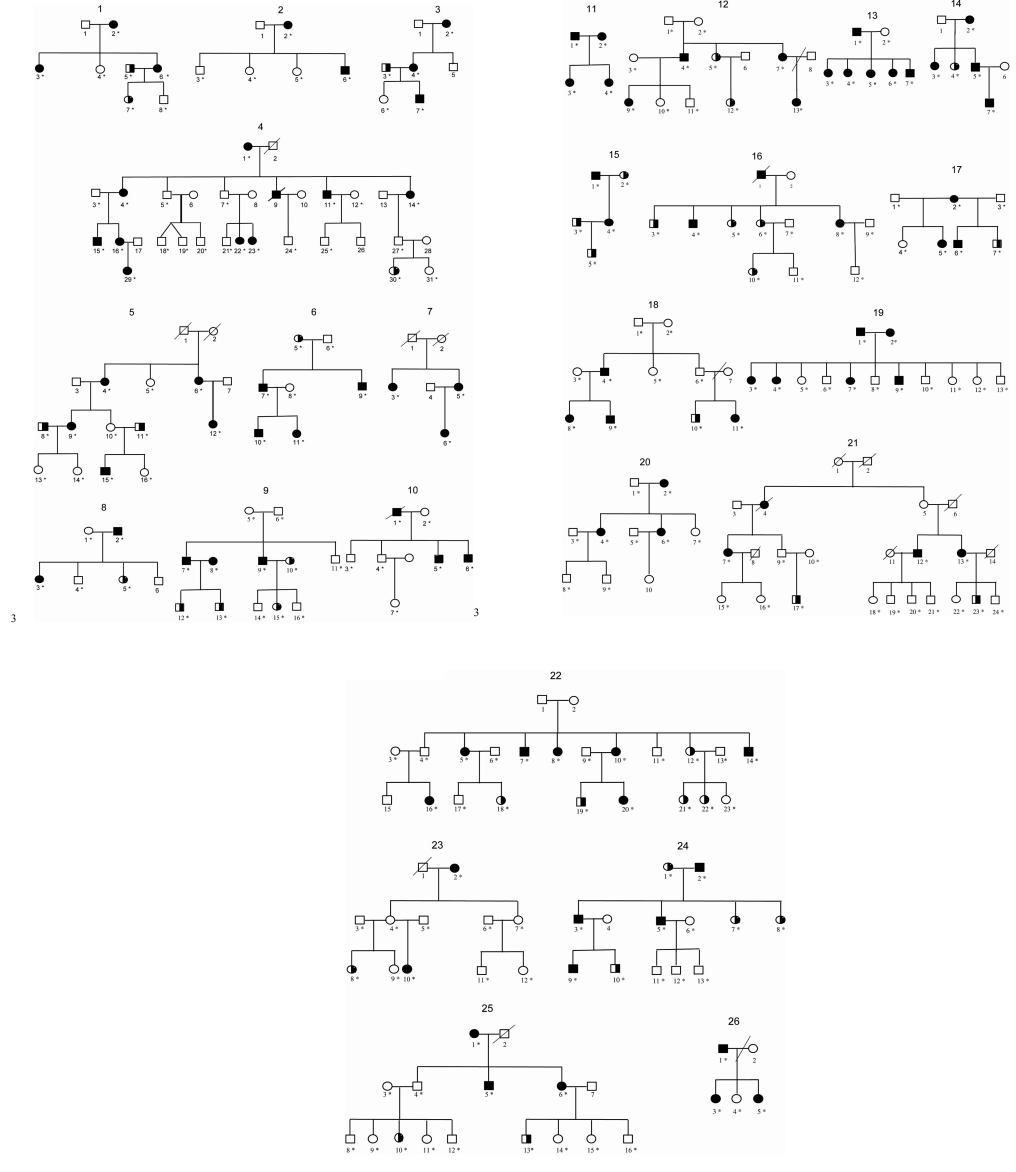
Genealogical trees of the 26 pedigrees. Black squares and circles denote subjects affected with high myopia (refractive value [RV] beyond −5 diopters [D]). Half-black squares and circles denote individuals with a RV between −0.5 and −5 D. The asterisks denote genotyped individuals.

A total of 26 families and their DNAs were collected from all over France. Twenty-five families were of French origin and one family (of 13 members) was of Algerian origin. The entire cohort included 347 individuals. The population characteristics are shown in Table 2 and Figure 1.

**Table 2:** Demographic characteristics.

**Demographic characteristics**	
Number of families analyzed	26
Number of individuals	347
Total number of affected individuals	98
Total number of low and moderate myopia	47
Total number of individuals genotyped	233
Average number of generations (range)	3.35 (2 to 4)
Average number of individuals per family (range)	13 (5 to 24)
Average number of affected individuals per family (range)	4 (2 to 10)
Average number of genotyped individuals per family (range)	9 (3 to 22)
Average age, in years, of examined individuals (range)	37 (5 to 95)
Average spherical equivalence (range)	−4.84±6.19 (−25 to 4.5)

A summary of the population characteristics is given including average data of the ophthalmological evaluations. Affected subjects are high myopes with refractive errors beyond −5 diopters (D). Low and moderate myopes have refractive errors between −0.5 D and −5 D. The mean values and the range of age variation (in years) and spherical equivalent (in diopters) are given

Clinical data were collected for the 233 genotyped individuals. Among them, 45% were men and 55% women. Age distribution was similar in both genders (78% of men and 75% of women were older than 20 years). Eighty-one adults over 20 years old (32 men and 49 women) and 17 patients under 20 years old (6 boys and 11 girls) had high myopia. The unaffected group was formed by 97 adults over the age of 20 years (49 men and 48 women) and 38 individuals under the age of 20 years (16 boys and 14 girls). No significant effect of sex (p=0.02), age (p=0.03), or ethnic origin (p=1) between affected and unaffected persons was observed.

### Linkage analysis of the nine new families

No significant or suggestive linkage was observed in parametric linkage (PL) and non-parametric linkage (NPL) analyses of the nine novel families in any model tested. No significant (p-value less than or equal to 0.000049) or suggestive (p-value greater than or equal to 0.000048 but less than or equal to 0.0017) [48] linkage.

### Linkage analyses in 26 families

**Figure 2:**
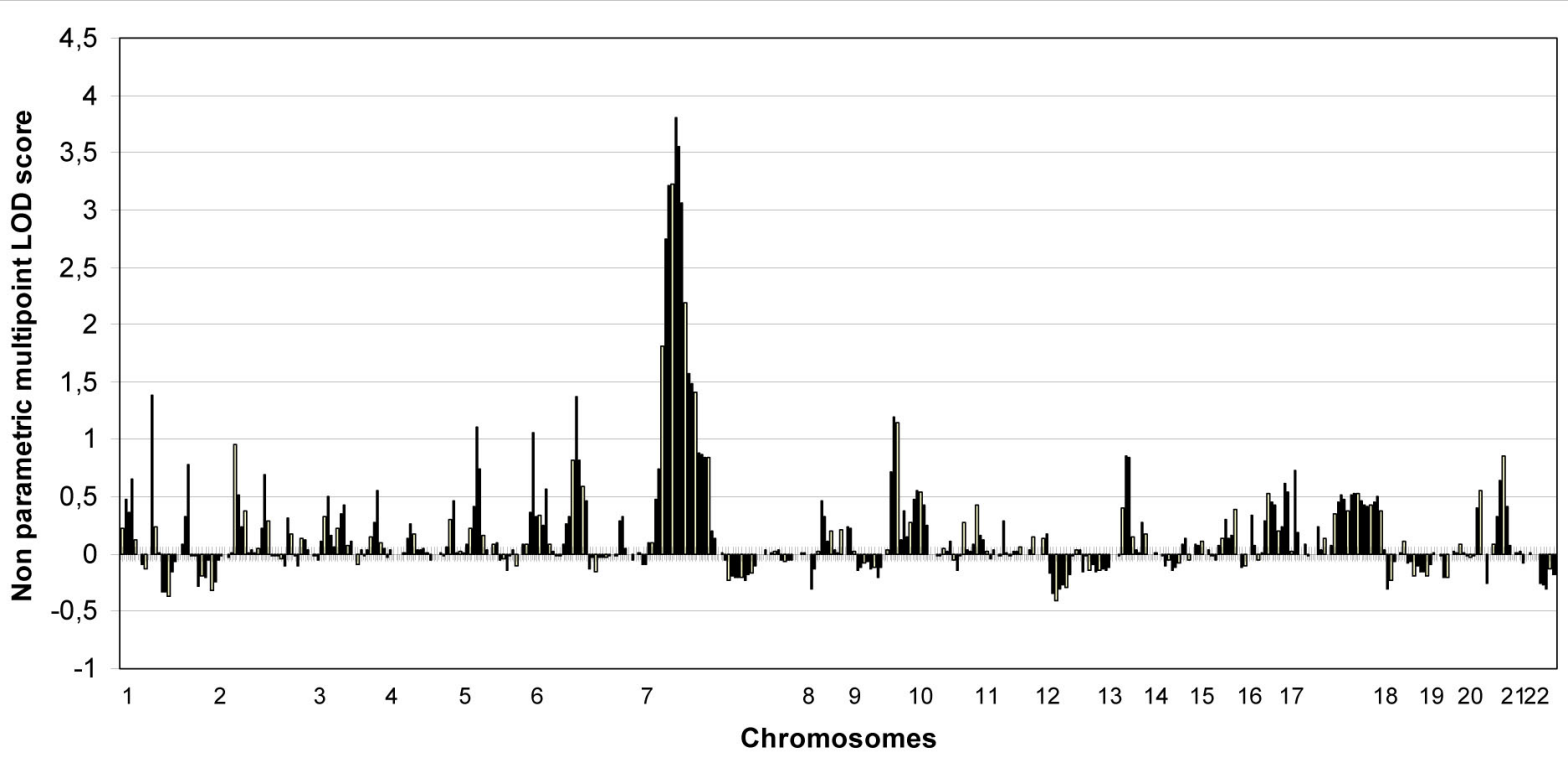
Non-parametric multipoint linkage analysis results for the 22 autosomes. Non-parametric multipoint linkage analysis results across the 22 autosomes. Genome-wide scan LOD scores of the entire population have been plotted against chromosomal location.

**Figure 3:**
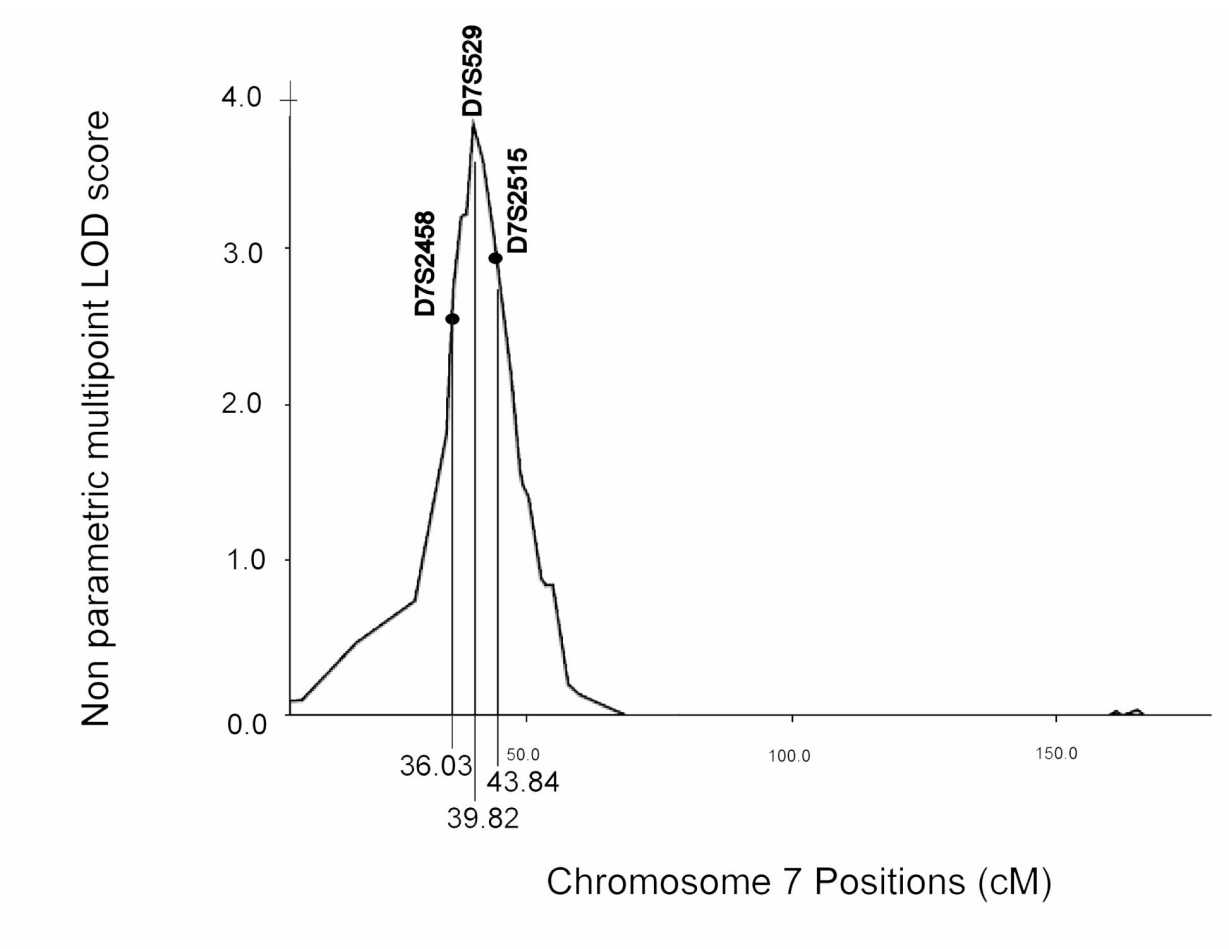
Non-parametric multipoint LOD score in 26 French families with myopia less than or equal to −5D for chromosome 7. LOD scores were plotted by Merlin 1.0.1 against marker distance given in centiMorgans (cM).

The PL analysis did not show any suggestive or significant linkage to any locus. Results of the NPL analysis are summarized in Table 3 and Figure 2. A significant NPL score of 3.74 (p=0.00002) and Z-NPL score of 4.07 (p=0.00002) was observed for markers D7S529 and D7S516 (Table 3 and Table 4). The region identified covers 7.81 cM between D7S2458 and D7S2515 (Table 4 and Figure 3). A weak signal was observed on chromosome 1p31, 6q15, and 9q21-q22 (Table 3).

**Table 3:** Maximum non-parametric multipoint linkage analysis results.

**Chromosome cytogenetic band**	**Number of markers on chromosome**	**Marker at the maximum NPL score**	**Position (cM)**	**Z-NPL**	**p value**	**NPL**	**p value**
1p31	31	D1S218	95.31	1.74	0.04	1.38	0.006
2p24	30	D2S305	38.87	1.18	0.12	0.95	0.02
3p21.1	23	D3S1277	61.52	0.94	0.2	0.65	0.04
4q34.1	22	D4S1539	176.19	1.30	0.1	0.46	0.07
5p15.1-p14.3	22	D5S416	28.76	1.84	0.03	1.11	0.012
6q15	33	D6S462	99.01	3.07	0.0011	1.37	0.006
7p15	49	D7S529	39.82	4.07	2.10**^−5^**	3.74	2.10**^−5^**
8p22	14	D8S549	31.73	0.83	0.2	0.46	0.07
9q21-q22	20	D9S287	103.42	2.32	0.01	1.20	0.009
10q26	20	D10S587	147.57	1.20	0.12	0.42	0.08
11p13	18	D11S935	45.94	1.21	0.11	0.29	0.13
12p13.2-q24.1	19	D12S83	75.17	0.31	0.4	0.03	0.4
13q14	14	D13S263	38.32	1.72	0.04	0.86	0.02
14q24.3	14	D14S74	87.36	0.41	0.3	0.13	0.2
15q23	14	D15S131	71.28	1.65	0.05	0.39	0.09
16q23.3	13	D16S3091	111.12	1.19	0.12	0.54	0.06
17q25.1	28	D17S1807	99.21	1.38	0.08	0.53	0.06
18q11.2-q12.1	14	D18S478	52.86	0.42	0.3	0.11	0.2
19q13.3	12	D19S902	72.72	0.63	0.3	0.09	0.3
20q13.13	13	D20S196	75.01	1.87	0.03	0.85	0.02
21q22	5	D21S266	45.87	0.13	0.4	0.01	0.4
Chromosome22	7	No positive hit					

An overview of the data obtained by the whole genome scan is provided with the number of markers used on each chromosome, marker chromosomal locations and their positions in centiMorgans (cM) on the genetic map. Markers were selected for each chromosome by the maximum non-parametric Z and LOD scores, which are associated with their p values.

**Table 4:** Non-parametric multipoint linkage analysis for region 7p15.

**Marker**	**Z NPL**	**p value**	**NPL score**	**p value**
D7S493	2.97	0.0015	1.68	0.003
D7S2458	3.5	0.0002	2.6	3x10^−4^*
D7S629	3.53	0.0002	3.05	9x10^−5^*
D7S673	3.53	0.0002	3.06	9x10^−5^*
D7S529	4.07	0.00002	3.74	2x10^−5^**
D7S516	3.97	0.00004	3.59	2x10–5**
D7S2515	3.31	0.0005	2.9	1.3x10^−4^*
D7S2496	2.73	0.003	2.9	1.1x10^−4^*
D7S632	2.22	0.013	1.42	0.005

Individual non-parametric Z and LOD scores are given with their p-values, for markers in the 7p15 region linked to high myopia. The asterisk indicates suggestive values and the double asterisk indicates significant values.

### Definition of high myopia phenotype

The definition of high myopia threshold is to some extent arbitrary. Thus, we examined the influence of the threshold used to define high myopia on the non-parametric results. Moderate myopic persons (with RV between −3 and −5 D) were considered affected in PL and NPL analysis. A suggestive linkage in 7p15.2–15.3 region was observed when the myopic threshold was equal to −6 D (p=0.0006), −3 D (p=0.00003), −2 D (p=0.00004), and −1.5 D (p=0.0006).

### QTL analysis on the 26 families

The RV mean for the entire cohort was considered for a QTL analysis but did not give any significant or suggestive linkage. Merlin 1.0.1 gave an estimation of heritability of 10% for the RV mean. The axial length was only available for 122 subjects with clinical examination, thus this trait was not used in the QTL analysis.

## Discussion

The aim of this study was to confirm previously reported linkage positions. The results of the genome wide scan (GWS) linkage study on the nine new families or on all 26 families together do not support the suggestive linkage to 7q36 region. The inclusion of new families and the enlargement of previously recruited families considerably modified the structure of the cohort. Although these data do not formally exclude the linkage of a high myopia locus on chromosome 7q36, it is likely that other myopia susceptibility loci play a more important role in the families that we studied. Our inability to confirm the previously identified position probably reflects the inclusion of more families with a larger sample size and of additional markers. Genotyping errors in the samples might vary between the study conducted by Naiglin et al. [28] and the current study. Such errors can influence linkage results by inflating recombination fraction estimates in linkage analysis. Replication of GWS studies for complex traits is difficult [48,49].

Another interesting finding is on chromosome 7p15 because linkage signal was detected in NPL analysis even when applying different definitions of the myopic phenotype. However, no significant linkage result in this region was found in any of the PL models used even under the hypothesis of genetic heterogeneity. Even if the NPL approach is robust in the face of uncertainty about the mode of inheritance [48,49], the new interval we have identified at 7p15.2-p15.3 needs to be confirmed. However, the same locus has also been highlighted in a Ashkenazi Jewish cohort [32]. Klein et al. [50] have also found a nearby region at 7p21.

The qualification of quantitative RVs as myopia, emmetropia, or hyperopia is to some extent arbitrary. A quantitative trait analysis was performed. This approach is generally more informative and powerful because continuous data on refractive errors (e.g., RV or axial length) provide more information than discrete trait data (e.g., affected and unaffected). In our cohort, the QTL analysis did not confirm the NPL result. The weak signal of the NPL analyses of binary traits may be due to low power. This paper highlights the complex heredity of high myopia and the great difficulty of replicating mapping results.

Sixty-seven genes or putative coding sequences are contained in the significant 7p15 region detected in this study (about 7.81 cM long). Candidate genes could be similar in function or structure to genes in other loci intervening in high myopia. Neurotransmitters can modulate experimental myopia [51]. A neuropeptide gene (*NPVF*) lies in our candidate region on 7p15.2-p15.3. The molecule encoded by this gene belongs to the family of neuropeptides with the Arg-Phe-amide motif at their C-termini (RF-amide peptides) involved in various neurotransmission/neuromodulation processes and muscle contraction control. It is expressed in the rat and human central nervous system [52] and in the retina [53]. Interestingly, it can inhibit GABAergic neurotransmission [54]. Gamma-aminobutyric acid (GABA) is an inhibitory neurotransmitter in adults but plays a neurotrophic role in the embryo. Stone et al. [54] have demonstrated that GABA receptor agonists and antagonists affect eye growth and modulate experimental myopia in chicken. The ability of NPVF to interact with the GABAergic system would be in accordance with both the strong effect of neurotransmitter modulation in experimental myopia and the role of heredity, which makes it a candidate gene for high myopia susceptibility. Similarly, other neurotransmitters that can be involved in experimental myopia such as neuropeptide Y (*NPY*) are encoded by genes present within the interval [55]. Recently, Han et al. [41] demonstrated the association of a hepatocyte growth factor (*HGF*) polymorphism with high myopia. The overexpression of *SNX15* leads to decreased cleavage of both insulin and HGF receptors [56], thereby highlighting a link between HGF and the sorting nexin family. Interestingly, our interval includes a member of the SNX family, *SNX10*. Further studies will be needed to determine putative association between such genes and the myopia phenotype within these families.
